# Protective efficacy of peptides from *Plasmodium vivax* circumsporozoite protein

**DOI:** 10.1016/j.vaccine.2020.03.063

**Published:** 2020-06-02

**Authors:** Erwan Atcheson, Arturo Reyes-Sandoval

**Affiliations:** The Jenner Institute, University of Oxford, Oxford, UK

**Keywords:** Vaccine, Circumsporozoite protein, *Plasmodium vivax*, Malaria, B-cell epitope, Virus-like particle, PvCSP, *Plasmodium vivax* circumsporozoite protein, PbCSP, *Plasmodium berghei* circumsporozoite protein, PfCSP, *Plasmodium falciparum* circumsporozoite protein, EU, Elisa Units, AI, Avidity Index, VLP, virus-like particle

## Abstract

Vivax malaria is a major cause of morbidity and mortality worldwide, with several million clinical cases per year and 2.5 billion at risk of infection. A vaccine is urgently needed but the most advanced malaria vaccine, VMP001, confers only very low levels of protection against vivax malaria challenge in humans. VMP001 is based on the circumsporozoite protein (CSP) of *Plasmodium vivax.* Here a virus-like particle, Qβ, is used as a platform to generate very high levels of antibody against peptides from PvCSP in mice, in order to answer questions important to further development of *P. vivax* CSP (PvCSP) vaccines. Minimal peptides from the VK210 and VK247 allelic variants of PvCSP are found to be highly protective as Qβ-peptide vaccines, using transgenic *P. berghei* parasites expressing the homologous *PvCSP* allelic variant. A target of neutralising antibodies within the nonamer unit repeat of VK210, AGDR, is found, as a Qβ-peptide vaccine, to provide partial protection against malaria challenge, and enhances protective efficacy when combined with full-length PvCSP vaccination. A truncated form of PvCSP, missing the N-terminal domain, is found to confer much higher levels of protective efficacy than full-length PvCSP. Peptides derived from highly conserved areas of PvCSP, RI and RII, are found not to confer protective efficacy as Qβ-peptide vaccines.

## Highlights

• Short repeat-region peptides from PvCSP on a VLP protect against malaria.
• The AGDR tetramer from PvCSP VK210 can, on a VLP, also protect against malaria.
• Full-length PvCSP is much less protective as a vaccine than truncated PvCSP.
• Region I and II peptides confer no protection against malaria presented on a VLP.

## 1 Introduction

Malaria caused by *Plasmodium vivax* causes several million clinical cases per year [1], with 2.5 billion at risk of infection [2], mainly in South East Asia and Latin America [3]. It is a highly neglected tropical disease; a vaccine would have enormous impact in control and elimination programs and is urgently needed [2], [3].

The leading vaccine candidate against *P. vivax* has recently been evaluated in a controlled human malaria infection study [4]. In that study soluble full-length *P. vivax* circumsporozoite protein (PvCSP) was used to vaccinate human volunteers. Low levels of protective efficacy were seen, prompting exploration of alternative strategies [5], [6], [7], [8]. The present study uses a virus-like particle, Qβ, as a platform for eliciting strong antibody responses against PvCSP peptides, followed by challenge of vaccinated mice with transgenic *P. berghei* parasites expressing the homologous PvCSP protein. By this means basic questions about the protective efficacy of B-cell epitopes within the PvCSP protein can be answered, and contribute to further development of PvCSP as a vaccine candidate.

The traditional target of neutralising antibodies in CSP is the central repeat region [9], [10]. In PvCSP two major allelic variants predominate, labelled “VK210” and “VK247” [11], [12]. Both are composed of a repeating unit of nine amino acids. A tetramer within this nonamer sequence, AGDR, found only in VK210, has been identified as a target of neutralising antibodies [13], [14], [15]. The NANP tetramer is a target of neutralising antibodies in PfCSP, but in that protein the repeat region is composed exclusively of such tetramers. No analogous tetramer within the PvCSP VK247 nonamer repeat has yet been identified. Epitopes outside of this central repeat region have been pursued as targets of neutralising antibodies, with limited success [16], [17], [18], [19], [20]. These regions, known as RI in the N-terminal domain and RII in the C-terminal domain, play functional roles in invasion of the liver by sporozoites [21], [22], [23], [24], [25], [26].

All three regions in CSP have been the subject, over many decades, of peptide-based vaccines designed to elicit neutralising antibodies, with limited success [14], [27], [28], [29], [30]. Until now these peptides have not been displayed on a highly immunogenic platform as a virus-like particle [31], [32]. Here for the first time this platform is used in efficacy testing of PvCSP peptides. For the first time also a vaccine based solely on the AGDR tetramer is tested for protective efficacy against homologous VK210 challenge.

## 2 Results

### 2.1 Immunogenicity and protective efficacy of PvCSP repeat peptides.

**Fig. 1:**
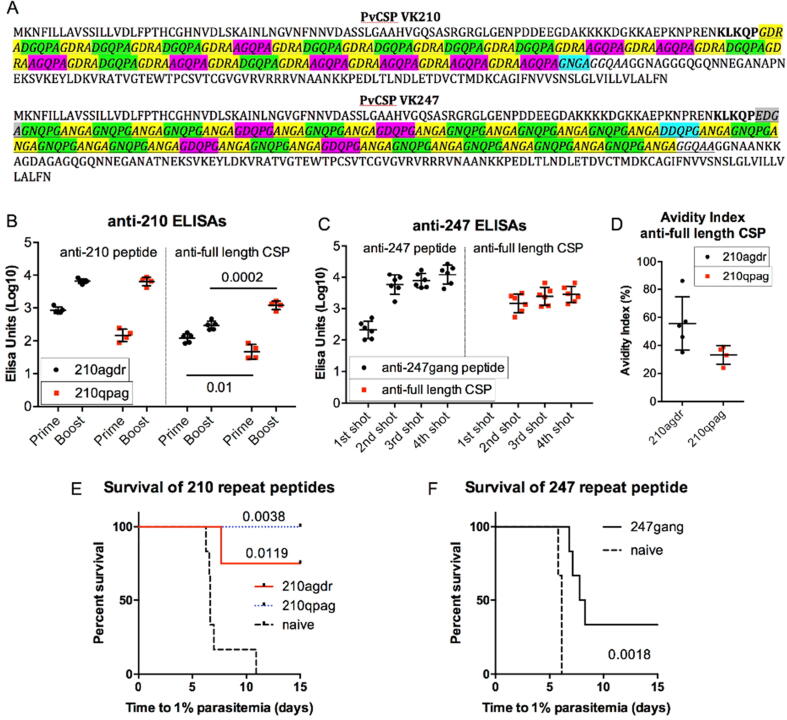
**Immunogenicity and protective efficacy of PvCSP repeat peptides.** (A) Representative *P. vivax* VK210 and VK247 sequences. BALB/c mice (n = 6) per group were vaccinated by intramuscular injection with peptides chemically coupled to Qβ virus-like particle (3 µg per dose) and delivered with Matrix-M™ adjuvant. Each peptide consists of two unit repeats of the central repeat region of *P. vivax* CSP VK210 (“210agdr” and “210qpag”), or VK247 (“247gang”). (B) Standard curve ELISAs for VK210 and (C) VK247 Qβ-peptide-vaccinated mice and (D) avidity indices for VK210 Qβ-peptide-vaccinated mice. Sera taken 2 weeks post-shot. Headings indicate peptide or protein used to coat ELISA plates. Means are shown ± SD. Numbers represent p-values from t-tests between indicated groups. Mice were challenged by intravenous injection of 1000 sporozoites and time to reach 1% blood-stage parasitaemia was calculated by linear regression from daily thin blood smears; mice were challenged with (E) PvCSP-210/PvTRAP *P. berghei* sporozoites for VK210 Qβ-peptide-vaccinated mice, and (F) for VK247 Qβ-peptide-vaccinated mice a PvCSP-247 replacement *P. berghei* parasite was used.

The primary structures of representative *P. vivax* CSP allelic variants are shown (Fig. 1A) with the central repeat regions and variations within those regions highlighted. Peptides consisting of two of these unit repeats were synthesized as shown in Table 1. The peptides were coupled to Qβ and used to immunize BALB/c mice. The Qβ-peptide vaccines each generated high-levels of antibody against their corresponding peptide as well as native full-length PvCSP protein (Fig. 1B, C). Interestingly 210agdr generated antibodies of higher affinity (by avidity index) to native PvCSP protein than did 210qpag (Fig. 1D), the sole difference being in the start-point of the repeat. Following challenge with transgenic *P. berghei* sporozoites with native PbCSP replaced by the corresponding allelic variant of PvCSP (homologous challenge), the VK210 Qβ-peptide vaccines conferred high levels of protection (100% (6/6 protected/challenged) and 83% (5/6) sterile protection for 210agdr and 210qpag respectively), and moderate protection conferred by 247gang (33% (2/6) sterile protection) (Fig. 1E, F). There was no clear association between avidity index and protection for the 210qpag-vaccinated group.

**Table 1:** Sequences of PvCSP repeat peptides.

210agdr	CGGDRADGQPAGDRADGQPAGDR
210qpag	CGGAGDRADGQPAGDRADGQPAG
247gang	CGGAGNQPGANGAGNQPGANG

### 2.2 Immunogenicity and protective efficacy of PvCSP non-repeat region peptides

**Fig. 2:**
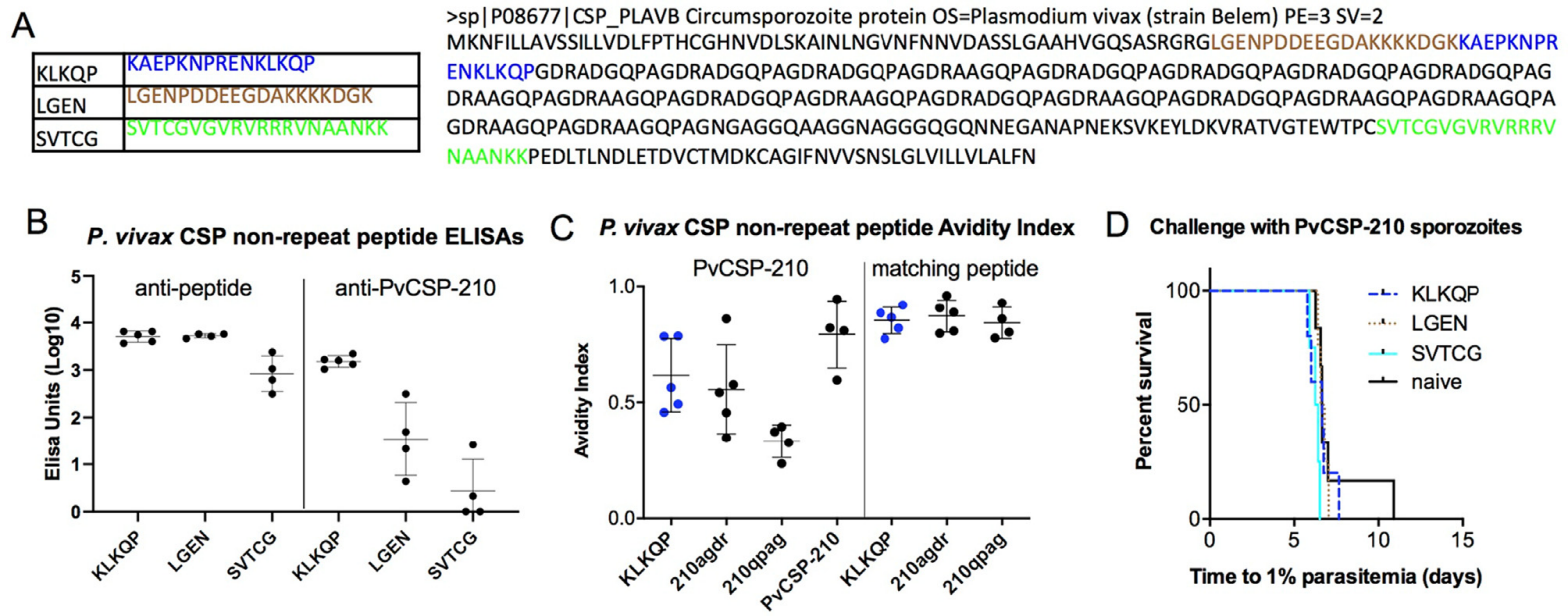
**Immunogenicity and protective efficacy of PvCSP non-repeat region peptides.** BALB/c mice (n = 4 to 6 per group) were vaccinated with non-repeat region PvCSP peptides chemically coupled to Qβ (3 µg per dose by intramuscular injection), using three-week intervals between shots with Matrix-M™ adjuvant. (A) The peptides used and positions in the *P. vivax* CSP primary structures are shown. (B) Standard curve ELISAs were performed using sera taken two weeks post-vaccination. (C) Avidity index represents the ratio of sera treated with 7 M urea to untreated sera in ELISAs. (D) Mice were challenged three weeks after the final shot with 1000 transgenic PvCSP-210/PvTRAP *P. berghei* sporozoites, and time to reach 1% blood-stage parasitaemia determined by linear regression using daily thin blood smears.

Having established that high levels of protective efficacy against homologous challenge could be conferred by Qβ-peptide vaccines displaying repeats from PvCSP, we asked whether peptides from non-repeat regions of PvCSP (Fig. 2A) could confer similar protection against challenge. Of three peptides so tested, all were highly immunogenic against the corresponding peptides (Fig. 2B, left-hand side). Only one (“KLKQP”), from Region I of PvCSP, generated antibodies capable of strongly recognising the native PvCSP protein (Fig. 2B, right-hand side), with affinity comparable to that of PvCSP-protein vaccinated mice, and higher than that of the highly protective PvCSP VK210 repeat-region Qβ-peptide vaccinated mice (Fig. 2C). Despite the quality of the Qβ-KLKQP peptides generated, however, neither the group vaccinated with this construct nor any other non-repeat region peptide conferred any level of protection against challenge (Fig. 2D).

### 2.3 Immunogenicity and protective efficacy of CSP repeat region tetramers

**Fig. 3:**
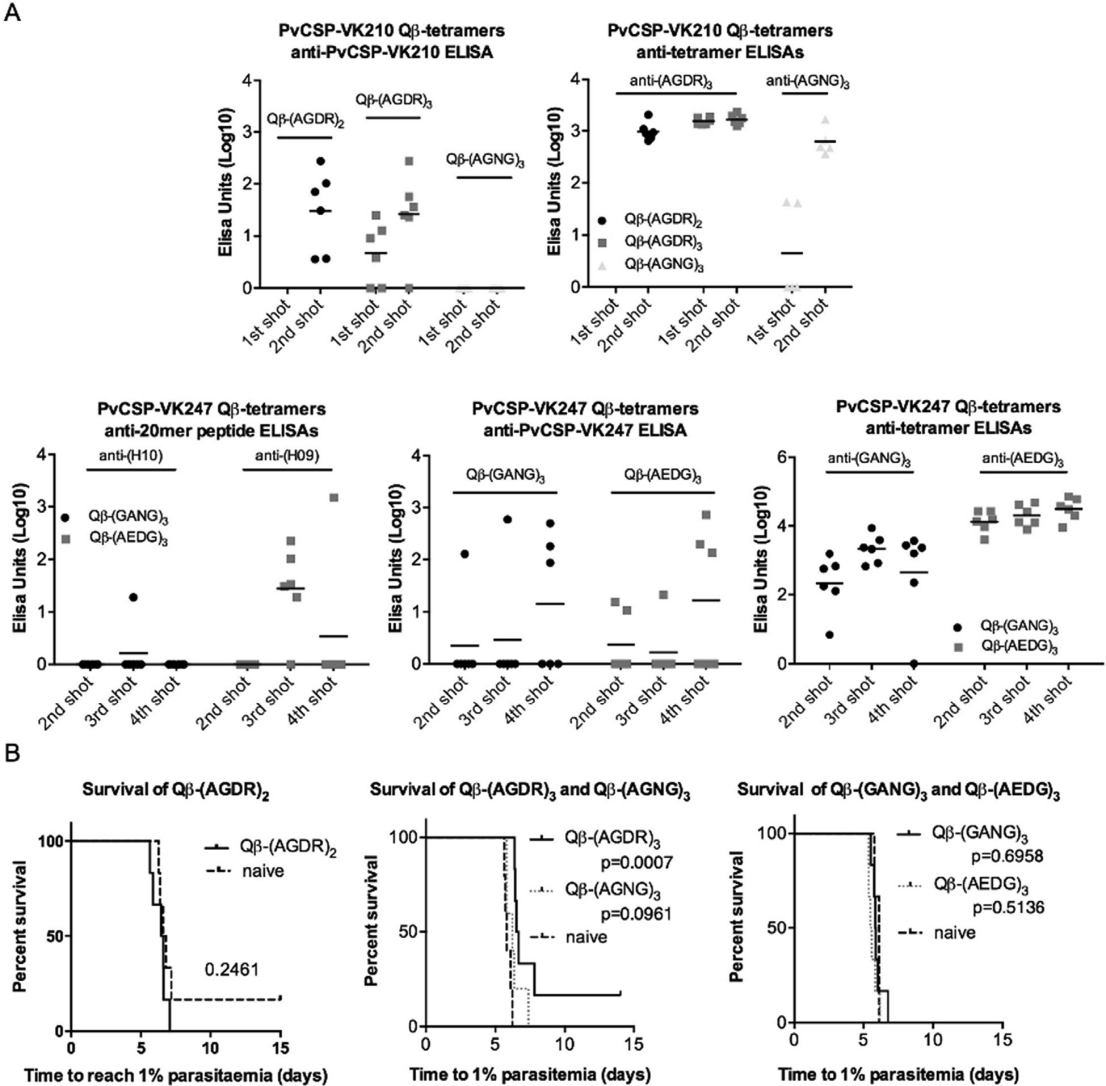
**Immunogenicity and protective efficacy of CSP repeat region tetramers.** BALB/c mice (n = 4–6 per group) were vaccinated by intramuscular injection with peptides consisting of 2, 3 or 6 copies of tetramers derived from *P. vivax* CSP VK210 or VK247, chemically coupled to Qβ virus-like particle and delivered with Matrix-M™ adjuvant. Mice were given two to four shots with a three week interval between shots. A dose of 3 µg Qβ-peptide per shot was used except with (AGDR)_2_ (8 µg per shot) and (AGDR)_3_ and (AGNG)_3_ (20 µg per shot). (A) Immunogenicity in ELISAs from plasma taken two weeks post-vaccination. ELISAs performed against indicated peptides or proteins. Peptides rom *P. vivax* CSP VK247: H09: GPEDGAGNQPGANGAGNQPG. *H*10: GANGAGNQPGANGAGNQPGA. (B) Mice were challenged three weeks after the final immunization by intravenous injection of 1000 transgenic *P. berghei* sporozoites: PvCSP-210/PvTRAP in the case of Qβ-(AGDR)_2_, Qβ-(AGDR)_3_, and Qβ-(AGNG)_3_; and PvCSP-247 in the case of Qβ-(GANG)_3_ and Qβ-(AEDG)_3_. Time to reach 1% blood-stage parasitaemia was calculated by linear regression using daily thin blood smears. P-values from Log-rank tests in comparison to naïves are shown.

Since a tetramer, AGDR, within the nonamer repeat unit of VK210 had previously been shown to be a target of neutralising antibodies, we were interested to test the protective efficacy of repeats of just this tetramer as a Qβ-peptide. Other tetramers within the VK210 and VK247 repeat region sequences were also synthesised and used as Qβ-peptide vaccines (Table 2). (AGNG)_3_ derives from AGNG as an equivalent tetramer to AGDR in a unique variant of the nonamer in the VK210 sequence. (GANG)_3_ derives from the GANG tetramer appearing to hold the same relative location within the canonical VK210 tetramer; and (AEDG)_3_ derives from EDGA possessing a similar position proximal to the RI region of PvCSP VK247 as AGDR does with VK210 (Fig. 1A). All Qβ-tetramer vaccines were highly immunogenic against their corresponding peptides but only (AGDR)-based vaccines generated antibodies recognising native PvCSP (Fig. 3A). Consequently only Qβ-(AGDR)_3_ vaccination conferred protective efficacy against homologous challenge (Fig. 3B).

**Table 2:** Sequences of PvCSP tetramer peptides.

(AGDR)_2_	CGGAGDRAGDR
(AGDR)_3_	CGGAGDRAGDRAGDR
(AGNG)_3_	CGGAGNGAGNGAGNG
(GANG)_3_	CGGGANGGANGGANG
(AEDG)_3_	CGGAEDGAEDGAEDG

### 2.4 Sequential and tandem immunisation with native PvCSP-210 and Qβ-(AGDR)_3_

**Fig. 4:**
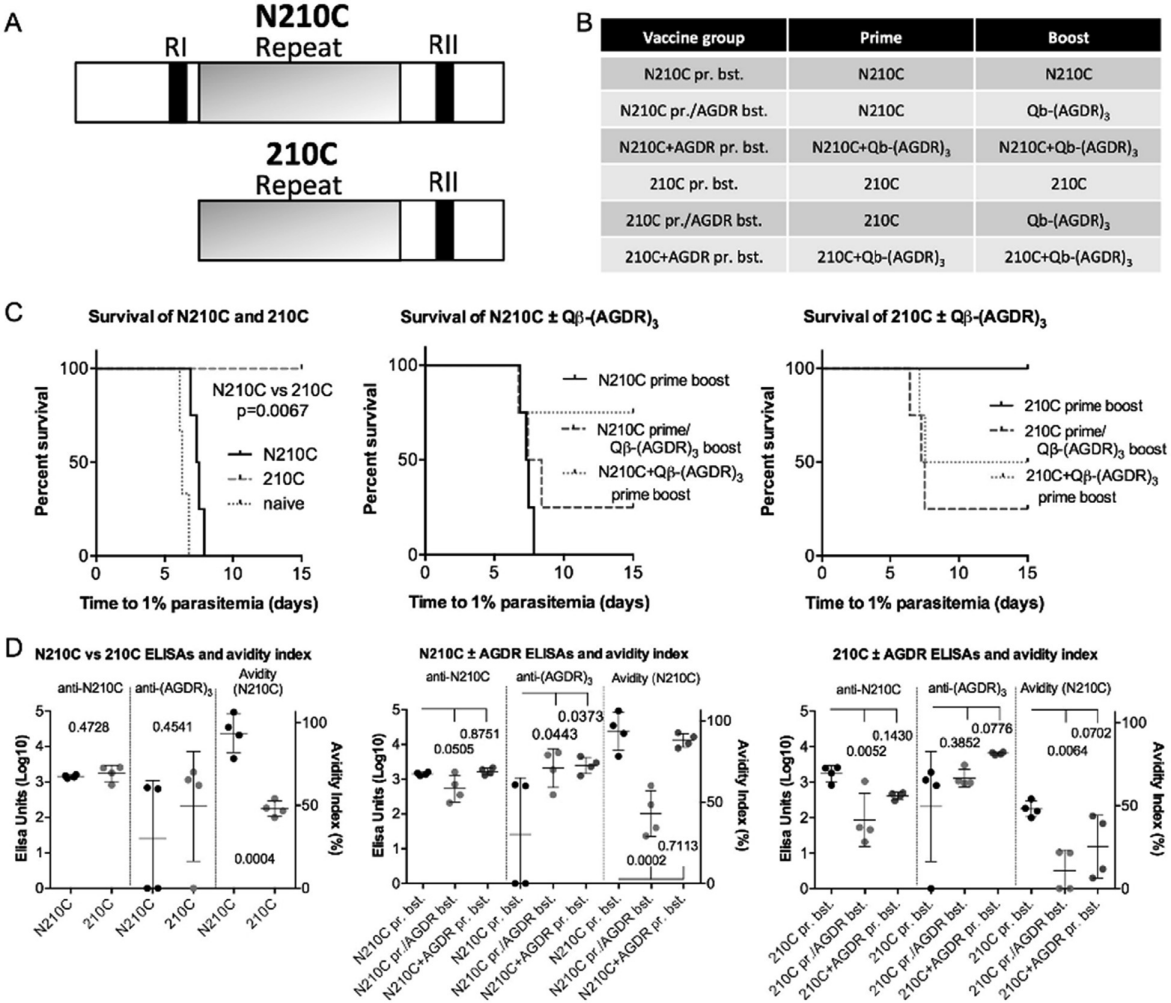
Sequential and tandem immunisation with native PvCSP-210 and Qβ-(AGDR)_3_. BALB/c mice (n = 4 per group) were immunized with full length (“N210C”) or truncated (“210C”) versions of PvCSP-210, as depicted schematically in (A). Some mice also received Qβ-(AGDR)_3_ in addition to either PvCSP-210 construct, or as a boost instead of the PvCSP-210, as shown in the table (B). Mice received 3 µg of each immunogen per shot with Matrix-M™ adjuvant, using a prime-boost regime with interval 3 weeks. 3 weeks after boosting, mice were challenged by intravenous injection of 1000 transgenic PvCSP/PvTRAP transgenic *P. berghei* sporozoites (C). P-value from comparison of N210C to 210C by log-rank test. (D) 2 weeks post-boost, serum was collected for mice for evaluation of immune responses by standard curve ELISA. Serum responses were tested against N210C and (AGDR)_3_ and avidity index determined by taking the ratio of ELISA units from serum treated or untreated with 7 M urea. Numbers represent P-values from t-tests (2 groups) or ANOVA with Tukey’s multiple comparison post-test (3 groups).

Having established that the AGDR peptide, as a Qβ-(AGDR)_3_ vaccine, could confer modest protective efficacy against challenge, we were interested to see whether combining this vaccine with native PvCSP vaccination could enhance the protective efficacy of the latter. CSP takes two forms, full-length and a truncated form missing the N-terminal domain; thus both full-length (N210C) and truncated (210C) versions were tested (Fig. 4A, B). Truncation of PvCSP markedly enhances its protective efficacy as a vaccine: 210C conferred 100% protection as against 0% protection for N210C (Fig. 4C). Combining Qβ-(AGDR)_3_ with PvCSP had very different consequences depending on whether it was the truncated form or not. Combining Qβ-(AGDR)_3_ with the full-length N210C improved protective efficacy, from 0% to 83% in the case of Qβ-(AGDR)_3_ given as a heterologous boost. Combining Qβ-(AGDR)_3_ with the truncated 210C, however, decreased protective efficacy (Fig. 4C).

To gain insight into the reason for the stark differences in challenge outcome depending on vaccination regime, standard and affinity ELISAs were performed (Fig. 4D). Full-length and truncated forms of PvCSP were equally immunogenic titred against native PvCSP, but N210C-vaccinated mice had much higher affinity against this form of the protein than did 210C-vaccinated mice. Combining the full-length N210C with Qβ-(AGDR)_3_ had no effect on recognition of the native PvCSP protein, but it did reduce affinity for the full-length PvCSP, particularly in the case of the highly protective N210C/Qβ-(AGDR)_3_ heterologous prime-boost regime, and anti-AGDR titres were increased. In contrast, combining truncated 210C with Qβ-(AGDR)_3_ produced a reduction in titres against the native protein, with the pattern of anti-AGDR and affinity otherwise similar to that obtained with full-length N210C combined with Qβ-(AGDR)_3_.

## 3 Discussion

Future vivax malaria vaccines will have to exploit every strategy available to maximise efficacy, given the failure of the most advanced vivax vaccine, VMP001, to deliver more than very low levels of protective efficacy [4]. Here, following proof of principle that Qβ-peptide vaccines can induce very high levels of protective efficacy, the platform was exploited to answer questions fundamental to further development of PvCSP as a vaccine candidate and to explore potential strategies for maximising efficacy. It was found that even with very potent antibody responses, Qβ-peptide vaccines based on RI and RII of PvCSP were not protective. A tetramer, AGDR, within the nonamer unit repeat of the central repeat region, however, did confer protection and was used in combination to enhance the protective efficacy of full-length PvCSP. Truncated PvCSP was found to confer much higher protection than full-length CSP, a finding that if validated could have important implications for CSP vaccine development.

The repeat region of CSP has long been known to be a target of neutralising antibodies [9], [10]. The first evidence that there were such targets outside of the CSP repeat region came from an experiment where sera from *Saimiri* monkeys immunized with PvCSP-VK210 vaccines were able to inhibit invasion of hepatocytes by *P. vivax* VK247 sporozoites [33]. The failure, here, of Region I and Region II peptides to generate neutralising antibodies is consistent with previous attempts (Table 3). This comes, in the present study, despite very high levels of antibody being generated and, in the case of the RI KLKQP-motif targeting vaccine, strong recognition of the native PvCSP. Although these are also known T-cell epitopes, here little T-cell response would have been engendered, as doses of 80 µg are required to elicit substantial T-cell responses [34]. The target should be pursued, however, as the epitope has been identified as a target of a potent neutralising monoclonal antibody, 5D5 [16]. The epitope is cryptic, not being recognised when presented within the full-length CSP [35]. Separate vaccination with the N-terminal region may succeed in eliciting neutralising antibodies. A vaccine consisting of the repeat region and the N-terminal domain only, and missing the C-terminal domain, might allow antibodies to be generated against Region I as then CSP will be in a potentially more immunogenic conformation [23]. If peptide-based Region I vaccines are to be pursued, extending the length of the peptide may be another good strategy for improving protective efficacy, as it has been found that deleting as few as three amino acids from either end of a 21 residue Region I peptide markedly decreases its ability to inhibit sporozoite invasion [35]. Interestingly, a one amino-acid change in the sequence of the PvCSP-VK210 repeat peptide used as a vaccine in the present study significantly affected immunogenicity and non-significantly reduced protective efficacy, so it is possible that the exact amino acid sequence, and not length, is the crucial parameter. However, the potently neutralising monoclonal antibody 5D5 can neutralize without recognising these extra amino acids [16]. It is possible that the epitope recognised by 5D5, though apparently linear, is in fact conformational, and that linear epitope mapping cannot detect other residues in the N-terminal domain it might bind to. A crystal structure of 5D5 binding the N-terminal domain would do much to clarify this, and would help inform vaccine design based on this epitope.

**Table 3:** Protective efficacy of CSP Region I and II peptides used as vaccines.

Region	Species	Sequence	Protective Efficacy/Notes	Ref
II	*P. falciparum*	EWSPCSVTCGNGIQVRIK	None against *P. berghei*	[17]
II	*P. falciparum*	IEQYLKKIKNSISTEWSPCSVTCGNGIQVRIK	80% in 100 sporozoite *P. berghei* challenge	[17]
II	*P. berghei*	GGNNNNKNNNNDDSYIPSAEKILEFVKQIRDSITEEWSQCNVTCGSGIRVRKRKGSNKKAEDLTLEDIDTEICKMDKCS	60% in 100 sporozoite *P. berghei* challenge	[20]
II	*P. falciparum*	KNNQGNGQGHNMPNDPNRNVDENANANSAVKNNNNEEPSDKHIKEYLNKIQNSLSTEWSPCSVTCGNGIQVRIKPGSANKPKDELDYANDIEKKICKMEKCS	No *in vitro* inhibition of HepG2 infection by *P. falciparum* or *P. berghei*	[20]
II	*P. falciparum*	TEWSPCSVTCGNGIQ	No *in vitro* inhibition of HepG2 infection by *P. falciparum*	[9]
II	*P. vivax*	SVTCGVGVRVRRRVNAANKK	None against 1000 PvCSP transgenic *P. berghei*	This study
I	*P. berghei*	GYGQNKSIQAQRNLNELCYNEGNDNKLYHVLNSKNGKIYIRNTVNRLLADAPEGKKNEKKNKIERNNKLK	50% in 100 sporozoite *P. berghei* challenge	[20]
I	*P. falciparum*	EYQCYGSSSNTRVLNELNYDNAGTNLYNELEMNYYGKQENWYSLKKNSRSLGENDDGNNEDNEKLRKPKHKKLKQPADGNPDPNANPNV	50% *in vitro* inhibition of HepG2 infection by *P. falciparum*	[20]
I	*P. falciparum*	DGNNEDNEKLRKPKHKKLK	Linear sequence recognised by potently neutralising 5D5 monoclonal antibody	[16]
I	*P. falciparum*	DKRDGNNEDNEKLRKPKHKKL	No reduction in liver burden	[19]
I	*P. falciparum*	KLKQPGDGNPDP	18% reduction in liver burden	[19]
I	*P. falciparum*	CKHKKLKQPGDG	No *in vitro* inhibition of HepG2 infection by *P. falciparum*	[9]
I	*P. vivax*	LGENPDDEEGDAKKKKDGK	None against 1000 PvCSP transgenic *P. berghei*	This study
I	*P. vivax*	KAEPKNPRENKLKQP	None against 1000 PvCSP transgenic *P. berghei*	This study
I	*P. falciparum*	EDNEKLRKPKH	None against 1000 PfCSP transgenic *P. berghei*	In press
I	*P. falciparum*	DDGNNEDNEKLRKPKHKKLKQPADGN	None against 1000 PfCSP transgenic *P. berghei*	In press

The AGDR epitope within the canonical PvCSP-VK210 nonamer unit repeat has received some attention from the vivax malaria vaccine community since a paper showed that *Saimiri* monkeys vaccinated with a recombinant PvCSP protein did not generate antibodies against this tetramer, and were not protected; but that a monoclonal specifically recognising AGDR was protective [13]. Responses to the AGDR tetramer were later associated with protection in *Saimiri* monkeys using a multiple antigen construct to deliver the nonamer epitope [14]. VMP001 [4] does not confer any sterile protection and generally fails to generate a detectable anti-AGDR response, while virus-like particle [5] or nanoparticle [36] display of VMP001 can, although in the former case 100% seroconversion was not obtained. Thus the protective efficacy seen here for the first time with Qβ-(AGDR)_3_-vaccinated mice, and the enhancement in protective efficacy it provides when used to boost full-length PvCSP-primed mice, is encouraging, validating AGDR-peptide based vaccination as a strategy for improving the protective efficacy of PvCSP-VK210-based vaccines. AGDR-peptide vaccination has been used before, with *Aotus* monkeys and BALB/c mice vaccinated with (AGDR)_6_ coupled to keyhole limpet hemocyanin [15]. Although mouse sera recognised the native PvCSP sequence and *P. vivax* sporozoites by immunofluorescence, that from *Aotus* monkeys did not. Thus it remains to be seen whether and how an AGDR-peptide based vaccination regime, alone or in heterologous prime-boost to ‘focus’ the immune response to this protective epitope, would enhance protection in humans.

No equivalent tetramer to AGDR was discovered here within the PvCSP VK247 nonamer repeat. The mechanism by which AGDR-specific antibodies mediate protection remains a mystery. One possibility is that such antibodies target the junctional region where cleavage occurs between the N-terminal domain and the central repeat region, a step essential to sporozoite infection of the liver [22], [23]: within PvCSP VK210 the tetramer GDRA lies exactly proximal to the conserved RI KLKQP sequence. Evidence that this may be the mechanism comes from the finding that a Qβ-peptide vaccine targeting this junctional region just downstream of KLKQP in PfCSP is protective (Atcheson et al, submitted).

There is a large difference in protective efficacy of 0% and 100% sterile protection respectively for full-length and truncated (missing the N-terminal domain) forms of PvCSP-VK210. Further validation of this finding should be a matter of priority, for it could explain the poor protective efficacy of VMP001 in clinical trial [4]**,** especially compared to the truncated form of PfCSP presented on RTS,S, highly protective in clinical trials [37]. Previously full-length PfCSP was found to be less immunogenic for the repeat region than truncated PfCSP [18]. In the present study, the difference in affinity of antibodies to the full-length PvCSP suggests that there may be a conformational difference between the full-length and N-terminal-truncated forms of PvCSP; structural studies will be required to verify this, and the possibility that crucial post-translational modifications are absent cannot be ruled out. The repeat region of PfCSP has been shown to possess multiple conformations [38], and thus it is possible that antibodies raised against the repeat region when it is in its full-length conformation do not bind and cannot neutralize the parasite when it displays CSP in its truncated conformation.

The Qβ-peptide platform has proven capable of eliciting high levels of protective efficacy against minimal epitopes from PvCSP. Further work could be done to test whether similar levels of protection are achieved with heterologous challenge as with, here, homologous PvCSP challenge. As with all pre-clinical studies, inference to clinic and field may be limited due to such factors as differences in virulance or infectivity of *P. berghei* spz compared to human malarias. However, pre-clinical models of leading malaria vaccines such as RTS,S have very often been consistent with subsequent clinical and field results [39]. The protective efficacy of an artificial (AGDR)_3_ peptide, not naturally present in the native PvCSP VK210 sequence, is capable of generating neutralising antibodies and enhancing protection with full-length PvCSP protein vaccination. RI and RII peptides are not protective as Qβ-peptide vaccines. These findings will help inform further development of CSP vaccines against vivax malaria.

## 4 Materials and methods

### 4.1 Vaccination

Isofluorane-anaesthetised mice were vaccinated by intramuscular injection (25G needle) of 25 uL vaccine formulation into left and right hind muscles, with three week prime-boost intervals between doses. Early experiments used higher doses (20 µg and 8 µg) but 3 µg was found sufficiently immunogenic; later experiments used this dose as standard. Direct comparisons of immunogenicity in this study are only between identical dosing regimens. Matrix-M™ adjuvant (Novavax AB, Uppsala, Sweden) was used at 12 µg per dose.

### 4.2 Mouse strains used

6 week-old female BALB/c (H-2^d^) mice were used for vaccination experiments, with age-matched controls. TO outbred mice and BALB/c mice were used for parasite maintenance and mosquito feeds. All mice from Harlan/Envigo.

### 4.3 Ethics statement

All animals and procedures were used in accordance with the terms of the United Kingdom Home Office Animals Act Project License. The procedures were approved by the University of Oxford Animal Care and Ethical Review Committee (PPL 30/2889 and P9804B4F1).

### 4.4 Infection of anopheles stephensi mosquitoes with *P. berghei*

Cryopreserved mouse blood stocks of wild type or transgenic *P. berghei* from liquid nitrogen were defrosted and immediately administered to naïve BALB/c or TO mice by intraperitoneal injection (100 µL). Thin blood smears were taken daily and when gametocytes were observed mice were anaesthetised by intramuscular injection (Rompun/Ketaset) for mosquito feed. Mosquitoes starved for 2 h were allowed to feed for 10–15 m on anaesthetised infected mice. Blood was taken from mice to confirm exflaggelation of gametocytes by microscopy. After feeding, mosquitoes were returned to fructose/P-amino benzoic acid on cotton wool and maintained in the Jenner Institute insectary (19–21 °C, 12 h light/dark cycle). One week after feeding a second feed was performed on an anaesthetised naive mouse to improve sporozoite yields. Mosquitoes were maintained for a total of 21 days prior to dissection of sporozoite-infected salivary glands.

### 4.5 Dissection of mosquito salivary glands and challenge of mice with sporozoites

21 days after feeding on *P. berghei* infected mice, mosquitoes were sedated at 4 °C for dissection. Salivary glands were dissected from mosquitoes under a microscope and removed by pipette into a glass tissue homogeniser containing 100 µL Schneider’s insect media with 10% FBS. Sporozoites were liberated from salivary glands by gently homogenising three times and counted using a haemocytometer. Sporozoite concentration was adjusted to 10^4^ sporozoites/mL for intravenous injection into the tail vein of mice of 100 µL (1000 sporozoites per dose, by insulin syringe).

### 4.6 Thin blood smears and calculation of time to reach 1% blood stage parasitaemia

Daily thin blood smears were prepared on glass slides from a drop of blood taken from the tail tip of challenged mice. Slides were fixed in methanol then stained in 5% Giemsa (Sigma) for 30 min and washed in water. 1000 red blood cells were counted for three to five consecutive days until the mouse reached 1% blood stage parasitaemia. Time to reach 1% blood stage parasitaemia was calculated by linear regression of log_10_ percentage parasitaemia against time post-challenge, as previously described [40]. Mice without parasites by day 15 were considered to have been conferred sterile protection against challenge.

### 4.7 Production of transgenic *P. berghei* parasites

*P. berghei* parasites expressing PvCSP VK210 (PVX_119355) and PvTRAP (XP_001614147.1) in place of endogenous PbCSP and PbTRAP [7], or PvCSP VK247 (Q7M3X0) in place of PbCSP [6], were produced as previously described.

### 4.8 Expression of proteins in HEK293 cells and purification

PvCSP protein was expressed as previously described [41]

### 4.9 Qβ virus-like particle production, purification and chemical coupling

Qβ virus-like particles derive from the *Escherichia coli* bacteriophage Qβ [42] and were prepared as previously described [32]. In brief, Qβ-transformed *E. coli* from glycerol stock was grown to 1 mL in LB/carbenicillin, then transferred to 1 L M9 media (with 2 mL MgSO_4_, 5 mL 40% glucose, 50 mL casamino acid, 500 µL vitamin B1, and 100 mg/mL carbenicillin) and incubated at 37 °C 250 rpm for 18 h. Cells were pelleted (4500 rpm, 25 min, 4 °C) and the supernatant discarded. The pellet was resuspended in PBS, centrifuged again (20 min, 14,000 g), and supernatant discarded. The pellet was lysed using lysis buffer (20 mM NaPO_4_ pH 7.5, 0.1% triton x-100, 5 mM EDTA, 100 U/g cells Benzonase, 10 µL/g cells Lysonase, 10 µL/ml protease inhibitor), and freeze/thawing the pellet in dry ice twice. Lysed cells were sonicated for 1 min (15 s on/30 s off, 30% intensity), centrifuged at 14,000*g* for 25 min, and the supernatant collected and filtered. Fractogel purification was carried out using 20 mM NaPO_4_ pH 7.2 buffer with either 150 mM or 1 M NaCl, followed by size exclusion chromatography. LPS levels were found to be very low.

Coupling Qβ peptides was performed by derivatising Qβ with reactive groups using succinimidyl-6-[(β-maleimidopropionamido)hexanoate] (SMPH) at 10X molar excess SMPH (1 h, 250 rpm RT), followed by three 1 m 100 kDa spin filtrations with PBS (Amicon 0.5 mL) to remove free SMPH. Peptides were synthesised with free cysteines rendering SATA derivation unnecessary. Peptides were incubated with SMPH-derivatised Qβ for 3 h (250 rpm, RT) and Qβ-VLPs stored at −20 °C. All peptides were synthesised by ThinkPeptides.

### 4.10 ELISAs: Standard curve, affinity

Nunc Maxisorp 96-well plates (Sigma) were coated with antigen (50 µL, 1 µg/mL in PBS) and incubated overnight at RT. Plates were washed 6 times with PBS/0.05% Tween (PBS/T) (Sigma) and blocked for 1 h with 10% skimmed milk (Sigma) in PBS/T (100 µL/well). Microvette serum tubes (Sarstedt) were used to collect blood from tail veins of mice and serum obtained by centrifugation (13,000 rpm, 10 min). Sera was typically diluted at 1:500 post-prime, 1:1000 post-second shot and 1:2000 post-third shot and applied to plates in triplicate after blocking (2 h RT incubation). Standard curves were prepared on each plate against antigen of interest by serial dilution of standard sera obtained by cardiac bleed from mice vaccinated with the specific antigen being tested in ELISA. Plates were washed as before and goat anti-mouse whole IgG alkaline phosphatase conjugate (Sigma) applied (50 µL/well, 1:5000 in PBS/T, 1 h RT). Plates were washed as before and 1 mg/mL pNPP (Sigma) in diethanolamine buffer (Pierce) applied to the plates (100 µL/well) and allowed to develop with readings on a BioTech Microplate Reader taken at 14 min and 1 h at 405 nm. Titres were expressed as arbitrary ELISA units (EU) relative to a standard curve.

To determine the avidity index, a replicate ELISA was performed identical to and simultaneously with the standard curve ELISA, except that after 2 h incubation with diluted sera, 100 µL 7 M urea (Sigma) was applied to each well for 10 min (excluding the standard curve). Plates were then washed and the ELISA completed as before. The avidity index is the ratio of urea-treated to untreated ELISA units, as previously described [43].

### 4.11 Statistical tests used

GraphPad Prism (MacOS v6) and Microsoft Excel were used for all statistical analyses performed. Student’s *t*-test and ANOVA with Bonferroni’s multiple comparisons test were used on parametric data comparing two or more groups respectively. Log-rank (Mantel-Cox) tests were used to determine significant differences between survival curves.

## Declaration of Competing Interest

The authors declare that they have no known competing financial interests or personal relationships that could have appeared to influence the work reported in this paper.
